# Analysis of open chromatin regions in bladder cancer links β-catenin mutations and Wnt signaling with neuronal subtype of bladder cancer

**DOI:** 10.1038/s41598-020-75688-0

**Published:** 2020-10-29

**Authors:** Aleyna Eray, Perihan Yağmur Güneri, Gülden Özden Yılmaz, Gökhan Karakülah, Serap Erkek-Ozhan

**Affiliations:** 1 Izmir Biomedicine and Genome Center, 35330 Balçova, İzmir Turkey; 2 Izmir International Biomedicine and Genome Institute, Dokuz Eylül University, 35340 Inciralti, Izmir, Turkey

**Keywords:** Bladder cancer, Data integration, Epigenomics, Epigenetics, Gene ontology

## Abstract

Urothelial carcinoma of the bladder is the most frequent bladder cancer affecting more than 400,000 people each year. Histopathologically, it is mainly characterized as muscle invasive bladder cancer (MIBC) and non-muscle invasive bladder cancer (NMIBC). Recently, the studies largely driven by consortiums such as TCGA identified the mutational landscape of both MIBC and NMIBC and determined the molecular subtypes of bladder cancer. Because of the exceptionally high rate of mutations in chromatin proteins, bladder cancer is thought to be a disease of chromatin, pointing out to the importance of studying epigenetic deregulation and the regulatory landscape of this cancer. In this study, we have analyzed ATAC-seq data generated for MIBC and integrated our findings with gene expression and DNA methylation data to identify subgroup specific regulatory patterns for MIBC. Our computational analysis revealed three MIBC regulatory clusters, which we named as neuronal, non-neuronal and luminal outlier. We have identified target genes of neuronal regulatory elements to be involved in WNT signaling, while target genes of non-neuronal and luminal outlier regulatory regions were enriched in epithelial differentiation and drug metabolism, respectively. Neuronal regulatory elements were determined to be ß-catenin targets (*p* value = 3.59e−08) consisting of genes involved in neurogenesis such as *FGF9*, and *PROX1*, and significantly enriched for TCF/LEF binding sites (*p* value = 1e−584). Our results showed upregulation of ß-catenin targets regulated by neuronal regulatory elements in three different cohorts, implicating ß-catenin signature in neuronal bladder cancer. Further, integration with mutation data revealed significantly higher oncogenic exon 3 ß-catenin mutations in neuronal bladder cancer compared to non-neuronal (odds ratio = 31.33, *p* value = 1.786e−05). Our results for the first time identify regulatory elements characterizing neuronal bladder cancer and links these neuronal regulatory elements with WNT signaling via mutations in β-catenin and its destruction complex components.

## Introduction

Bladder cancer is one of the most frequent cancers and results in more than 150,000 deaths each year, constituting a major burden for the human health^1^. Over 90% of bladder cancers originate from urothelium and are called as urothelial bladder cancer. Histopathologically, bladder cancer is classified as non-muscle invasive bladder (NMIBC) and muscle invasive bladder cancer (MIBC)^2^. Though NMIBC has a better prognosis compared to MIBC, NMIBC also requires long-term follow-up and roughly 15% of these patients also progress into MIBC^3^. Current treatment options both for NMIBC and MIBC are rather unspecific, largely consisting of transurethral resection of bladder, BCG application and chemotherapy for NMIBC and mainly radical cystectomy and chemoradiotherapy for the treatment of MIBC patients^4^. Over the last decade, there have been several studies which aimed to identify the mutational landscape and molecular subtypes of bladder cancer^5–8^. These studies provided information about the frequently mutated genes implicated in bladder tumorigenesis and defined molecular subtypes based on gene expression.

Almost all the studies characterizing the mutational landscape of urothelial bladder tumors pointed out to the high frequency of mutations in genes involved in chromatin regulation, implying the deregulation of epigenetic mechanisms in bladder cancer. Despite these findings, the studies investigating the epigenetics of bladder cancer are mostly limited with DNA methylation profiling and identification of several genes with altered levels of DNA methylation^7^. Nevertheless, there have not yet been a study characterizing the epigenomic regulation in bladder cancer in detail.

Recently, largely the studies performed by ENCODE and Roadmap consortiums^9,10^ identified the epigenetic landscape of various cancer cell lines and normal tissues which enabled annotation of genomic regions implicated in the regulation of diverse tissue types. These studies served as a model for the design of the studies which aimed to identify epigenomic landscapes in primary cancer tissues. In this regard, the studies which characterized epigenomic regulation in pediatric brain tumors^11–13^, and hematological malignancies^14^ provided important insights about the transcriptional regulatory characteristics of these tumors.

A recent study identified pan-cancer active regulatory elements via involving 410 primary cancer tissue samples used in TCGA across 23 cancer types using ATAC-seq, a robust technique to reveal active regulatory regions in the genome^15^. In this study, the authors included 10 MIBC tumor samples originally described in TCGA study. Although over 500,000 transposase accessible regulatory elements were identified and explored in this study, the study followed a ‘pan-cancer’ analysis strategy except breast cancer, without focusing on the molecular subtypes of MIBC.

In this study, we thoroughly re-analyzed ATAC-seq data generated for MIBC^15^ and identified MIBC subtype specific regulatory signatures via integrating this data with DNA methylation and gene expression profiling identified for the respective samples. Annotating the MIBC samples with the molecular subgroups defined in TCGA study, we defined three MIBC subgroup specific active regulatory regions, which we termed as neuronal, non-neuronal and luminal outlier. Integrating our findings with gene expression and DNA methylation data available for all MIBC patients in TCGA consortium, we were able to link the mutational landscape of neuronal bladder cancer with regulatory regions specific to this subtype.

## Methods

The analyzes used for this manuscript were done using the R programming language^16^.

### Data acquisition and preprocessing

All the data used in this manuscript is publicly available and has been downloaded from the websites/databases indicated below.

#### ATAC-seq

ATAC-seq peaks called for bladder cancer (n = 10) and normalized ATAC-seq signal for the respective peaks^15^ were obtained from the TCGA Publication Page link (https://gdc.cancer.gov/about-data/publications/ATACseq-AWG).

#### DNA methylation

TCGA-BLCA 450k DNA Methylation data provided by^7^ was used. Normalized DNA Methylation beta values of 412 bladder cancer patients have been obtained via “TCGAbiolinks” R Bioconductor package.

#### Gene expression

Gene expression data provided by^7^ was obtained via “TCGAbiolinks” R Bioconductor package available for 408 bladder cancer patients. FPKM-UQ data was used for gene expression analysis. Log2 normalization applied to the gene expression data.

Gene expression data provided by^17^ was obtained via “consensusMIBC” R package^18^. Robust Multichip Average (RMA) data was used for gene expression analysis.

Gene expression data provided by^19^ was obtained via cBioPortal database. Quantile Normalization (QN) data was used for gene expression analysis.

ß-catenin target gene expression analysis and visualization were performed by calculating the mean expression values of the genes for neuronal and non-neuronal subgroups within each cohort (Fig. 4c–e).

### Data analysis and integration

#### ATAC-seq data analysis and clustering

Bladder cancer ATAC-seq signals at all the MIBC ATAC-seq peaks (n = 107,921) were averaged between the replicates of the ATAC-seq MIBC cohort (n = 10). Afterwards, to obtain the most variable 20,000 ATAC-seq peaks, “sd” function -which calculates the standard deviation among given numbers- was applied to the data by using R programming language. To cluster the retained 20,000 peaks, “kmeans” function of the “stats” package of R was used. Clustering was performed by using k means with k = 3, empirically chosen such that the analysis resulted in distinct clusters consisting of comparable regulatory elements. Annotations of the MIBC were done according to the mRNA subgroup definitions from TCGA study^7^.

#### Integration with DNA methylation data

As DNA methylation beta-values were based on hg19 assembly, we first lifted the coordinates of the regulatory clusters to hg19 using USCS liftOver tool^20^. With this liftover, 4854 (cluster 1), 8035 (cluster 2), 7028 (cluster 3) regulatory regions were retained for further analysis. Afterwards, for each of the regulatory clusters we defined (neuronal, non-neuronal and luminal outlier), genomic coordinates of the respective regulatory cluster were overlapped with the 450k CpG coordinates using “GenomicRanges” R Bioconductor package. Neuronal cluster, non-neuronal cluster and luminal outlier clusters overlapped 1229, 3846 and 1667 CpG sites, respectively. Afterwards, average DNA methylation levels for each MIBC tumor (n = 412) were determined by calculating the average beta-value at the CpG sites overlapping with the respective regulatory cluster. For each regulatory cluster, average beta-values were represented as boxplots according to the molecular subgroup annotations from^7^. Statistical testing of the differences in DNA methylation levels across the subgroups was performed using ANOVA and post hoc tests.

#### Identification of target genes of MIBC subgroup specific active regulatory regions

For the regulatory elements present in the three clusters we determined, we investigated whether the target genes of the respective regulatory elements are already defined in the study from^15^ based on the pan-cancer ATAC-seq signal vs gene expression correlation analysis (Supplementary Table 7 from^15^) Supplementary Table 7 from^15^ provides pan-cancer peak ids, their genomic coordinates and their linked target genes. For the ATAC-seq peaks we used in clustering analysis (Fig. 2a), we matched the peak ids with the ones available in Supplementary Table 7 from^15^ and in this way identified the target genes of the three regulatory clusters we defined (see Supplementary Table S1 online).

#### Go term and pathway analysis

For GO term and pathway analysis, “clusterProfiler”^21^ R Bioconductor package was used. For both KEGG pathway and GO term analysis, only the terms below 0.05 FDR threshold were included. GO term analysis was performed for only ‘biological process’.

#### Transcription factor analysis and transcription factor motif finding

To explore which transcription factors are involved in the regulation of the genes targeted by neuronal regulatory elements, online database Enrichr^22,23^ with the ‘transcription ChEA’ option was used (Fig. 3a). Transcription factor motif finding at the regulatory elements defined in^24^.

Figure 2a was performed via HOMER motif finding algorithm^24^ using the “findMotifsGenome.pl” command with -size given and hg38 options.

#### Mutation analysis

For mutation analysis of bladder cancer patients^7^, cBioPortal database was used. ß-catenin and the genes which are the members of ß-catenin destruction complex were investigated for their mutation rate for both neuronal and non-neuronal samples. To see the effects of these mutations on proteins, DANN, SIFT and PROVEAN scores were used. MutationTaster database was used to predict how the mutation affects the protein's function.

### Data visualization

#### Heatmap representations

ATAC-seq signal of the clusters defined by k-means was visualized using ‘pheatmap’ function of pheatmap R package^25^ (Fig. 2a).

#### Boxplot and dotplot representations

To represent boxplots, R “boxplot” function was used. For visualization of the pathway enrichment analysis, “dotplot” function of “enrichplot” R package^26^ was used.

#### Snapshot representations

USCC Xena browser^27^ was used to visualize the data all for ATAC-seq, DNA methylation and gene expression available for 10 samples. ATAC-seq peak regions and the specific gene regions that are linked to those peak regions were visualized (Fig. 4a,b).

#### Oncoprint images

Oncoprint images to represent the mutation rates in ß-catenin and ß-catenin destruction complex components were generated via https://www.cbioportal.org.

### Statistical analysis

Statistical analyzes were done by using R/Bioconductor packages (www.bioconductor.org). In boxplot comparisons through in Fig. 4, ANOVA test was used to test the statistical difference among the bladder cancer subtypes. Post hoc tests after ANOVA was performed using “PostHocTest” function of “DescTools” R package^28^. In Fig. 5, Wilcoxon rank sum test was used to test the statistical difference between neuronal vs non-neuronal bladder cancer. Significance threshold was set as *p* value < 0.05. To see the enrichment pattern of ß-catenin in neuronal subtype versus non-neuronal bladder cancer, Fisher’s Exact test was used (Fig. 6).

## Results

### MIBC subgroup specific regulatory regions and their target genes

**Figure 1:**
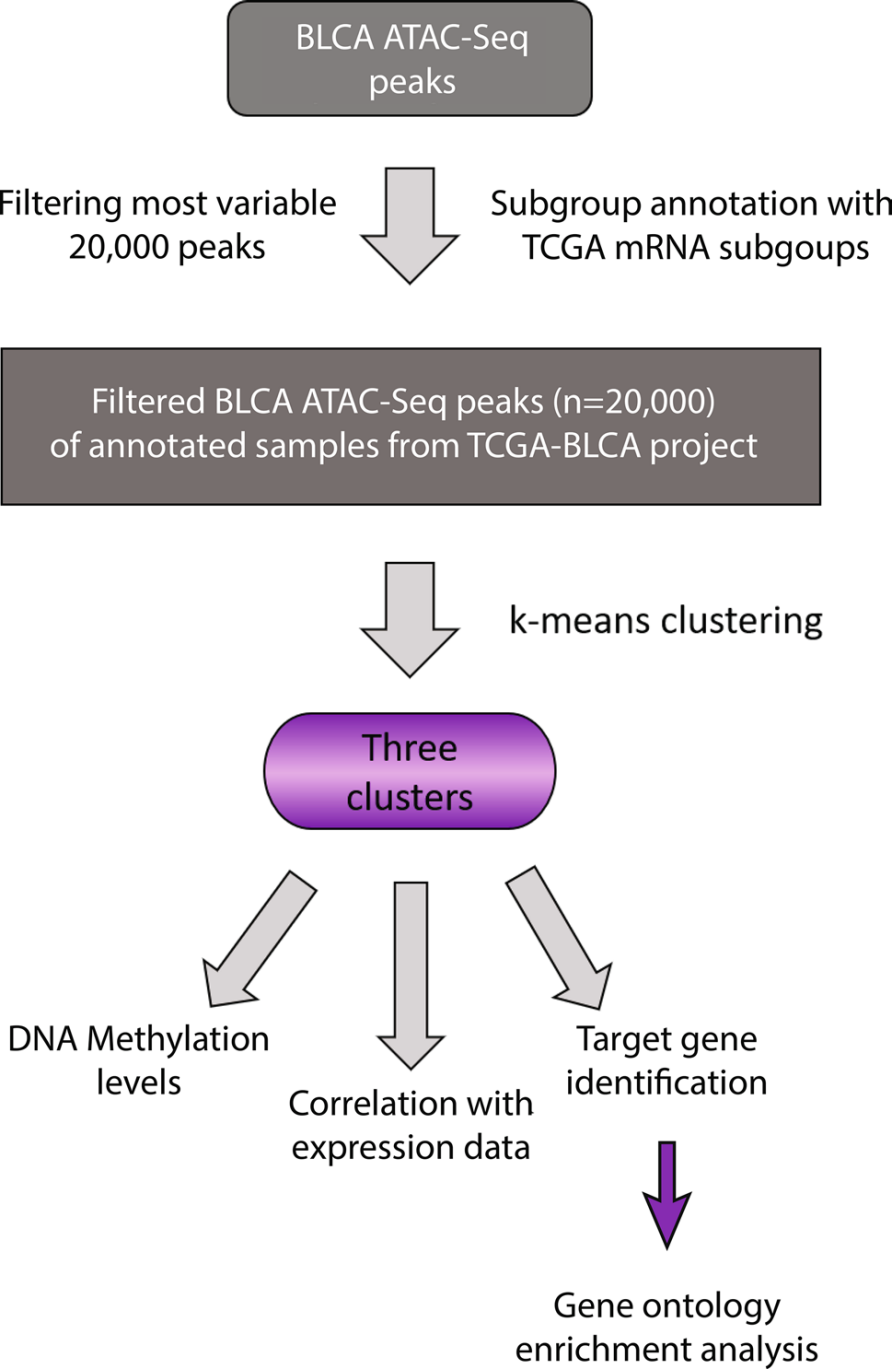
Flowchart of data processing. The flowchart shows the steps of data processing and integrative data analysis.

**Figure 2:**
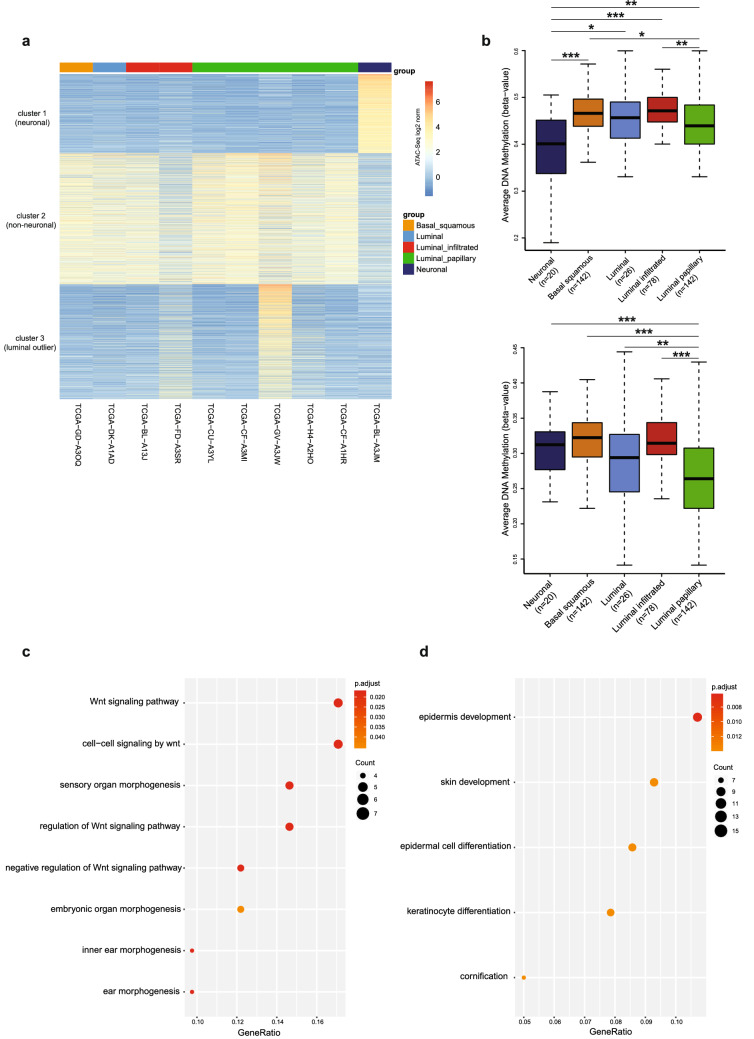
Subgroup specific regulatory regions of bladder cancer. (**a**) Heatmap visualization of the k-means clustering of most variable bladder cancer ATAC-seq peaks. Color annotations refer to mRNA subgroups identified in the study^7^. Three clusters were represented; cluster 1: neuronal, cluster 2: non-neuronal and cluster 3: luminal outlier. (**b**) DNA methylation levels (Beta values) of mRNA subgroups on the regulatory regions: cluster 1 (upper panel) (Anova *p* value = 1.91e−07) and cluster 2 (lower panel) (Anova p value < 2e−16). PostHocTest significance codes:*p < 0.05; **p < 0.01; ***p < 0.001. (**c**,**d**) Gene Ontology terms enriched for the target genes of cluster 1 (**c**) and cluster 2 (**d**). The dotplot was generated using “clusterProfiler” R/Bioconductor package. Dot sizes indicating gene counts of specific terms.

We re-analyzed and clustered chromatin accessible regions defined for bladder cancer from^15^ (Fig. 1). Using subgroup specific annotations from latest TCGA study^7^ enabled us to define three bladder regulatory regions, which we termed cluster 1 (n = 4868) as neuronal, cluster 2 (n = 8067) as non-neuronal and cluster 3 (n = 7065) as outlier luminal (Fig. 2a). To understand the subgroup specificity of the regulatory regions we defined, we calculated DNA methylation levels on the identified clusters using 450k DNA methylation data, available for 412 bladder cancer patients. In line with the regulatory regions we defined, bladder cancer patients belonging to neuronal molecular subtype showed the lowest DNA methylation levels at the neuronal regulatory regions (cluster 1), (*p* value = 1.91e−07). For the non-neuronal regulatory regions, neuronal subtype was significantly hypermethylated compared to other subtypes, (*p* value < 2e−16) (Fig. 2b). For cluster 3, which we annotated as luminal outlier, DNA methylation levels for the molecular subtypes varied (see Supplementary Fig. S1 online). However, comparing the DNA methylation level of the outlier luminal papillary sample (TCGA-GC-A3JW) versus the other 4 luminal papillary samples included in the clustering analysis revealed that DNA methylation level of the outlier sample was significantly lower compared to other luminal papillary samples at cluster 3 regulatory elements (see Supplementary Fig. S1 online). These findings suggest that regulatory region classifications we defined for bladder cancer are in good concordance with the DNA methylation landscape of the respective regions, meaning that we identified lower DNA methylation levels for a molecular subgroup at the active regulatory elements belonging to the same subgroup over the whole TCGA cohort (n = 412).

Once the regulatory elements are defined, one of the key questions is to link the regulatory elements with their target genes. Although several strategies have been employed to address this question, it appeared that identification of the target genes based on correlation of regulatory region signal activity and expression level of genes gives very reliable results^29,30^. Corces et al. followed a similar strategy to define the target genes of DNA regulatory elements they defined pan-cancer. In order to determine the genes and pathways differentially implicated for the three clusters we defined (Fig. 2), we checked whether the three regulatory clusters we defined for bladder cancer were linked to target genes in^15^. Employing this strategy, we determined 117 target genes for neuronal cluster, 371 target genes for non-neuronal cluster and 107 target genes for luminal outlier cluster (see Supplementary Table S1 online).

Remarkably, target genes of the neuronal cluster were exclusively involved in WNT signaling and its regulation (Fig. 2c). Association of the neuronal subtype with WNT signaling was not a finding which was reported in the latest TCGA cohort study^7^. Only one study, which re-analyzed gene expression data over 35 cohorts belonging to 2,411 NMIBC and MIBC tumors showed the up-regulation of genes involved in WNT signaling in the neuronal subtype among the 6 molecular subtypes defined in the same study^8^ without providing mechanistic details. Neuronal molecular subtype of bladder cancer was previously associated with neuronal differentiation pathways^7,17^. In our analysis, although ‘neurogenesis’ and associated pathways are not directly highlighted in Fig. 2c, we identified 9 genes to be involved in neurogenesis including *CNTN1*, *FOXD1*, *PROX1*, *NKD1*, *SOX5*.

Performing a GO term enrichment analysis for the non-neuronal cluster target genes revealed an enrichment for the terms epithelial differentiation and epidermis development, suggesting divergence of non-neuronal bladder cancer from the neuronal ones mainly by epithelial characteristics (Fig. 2d). Although we could not identify a gene ontology biological process significantly implicated for the luminal outlier cluster, performing a KEGG pathway analysis for this cluster showed an enrichment for Drug Metabolism and Chemical Carcinogenesis Pathways (see Supplementary Fig. S2). Following upon this finding and inspecting the available clinical data, we identified that this outlier patient received the Carboplatin and Gemzar combined chemotherapy^7^. Among the other 9 bladder cancer patients we analyzed, only 2 of them had available clinical data, and both received Gemcitabine (Gemzar) and Cisplatin therapy (see Supplementary Table S2 online), implying that treatment with Carboplatin might have an effect on the differential regulatory landscape of this outlier sample belonging to the luminal subtype. However, this result should be interpreted with caution because of the lack of sufficient data.

### Neuronal active regulatory regions are ß-catenin and TCF/LEF targets

**Figure 3:**
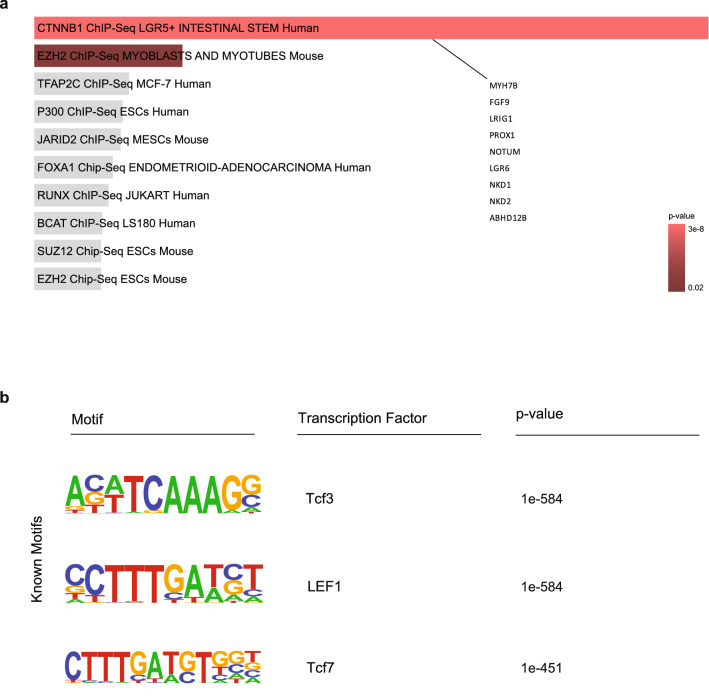
Transcription factor motif analysis for the neuronal regulatory elements. (**a**) ChEA factor analysis using the program EnrichR for the target genes of the neuronal regulatory elements (*p* value = 3.59e−08). (**b**) HOMER known transcription factor motif analysis at the neuronal regulatory regions. Top three enriched motifs are displayed.

As we identified WNT signaling to be associated with neuronal specific regulatory elements, we asked whether neuronal regulatory elements were enriched for certain transcriptional factors. Initially, performing an analysis via EnrichR program significantly associated the target genes of the neuronal regulatory elements with ß-catenin targets (*p* value = 3.59e−08) (Fig. 3a, see Supplementary Table S3 online). Afterwards, we performed an unbiased transcription factor motif enrichment analysis at all neuronal specific regulatory elements (Fig. 2a, cluster 1) independent of their association with a gene target. Remarkably, this analysis identified the TCF/LEF binding motifs as the top enriched motifs in both de novo and known motif finding modes of the HOMER algorithm^24^ (Fig. 3b, see Supplementary Fig. S3, see Supplementary Table S4 online). In comparison, transcription factor motif analysis for non-neuronal regulatory regions did not show an enrichment for TCF/LEF motif (Data not shown). TCF/LEF transcription factors which bind ß-catenin directly and regulate the expression of target genes are the major downstream factors of WNT signaling in the nucleus^31,32^. ß-catenin itself does not have a binding motif available in the HOMER algorithm as it is a transcriptional co-activator^33,34^. However, having identified the motifs of TCF/LEF, which can directly bind ß-catenin, directly at neuronal regulatory elements highly argues for the regulation of neuronal regulatory regions by ß-catenin-TCF/LEF complex.

### Target genes of neuronal regulatory elements regulated by ß-catenin are overexpressed in neuronal subtype bladder cancer

**Figure 4:**
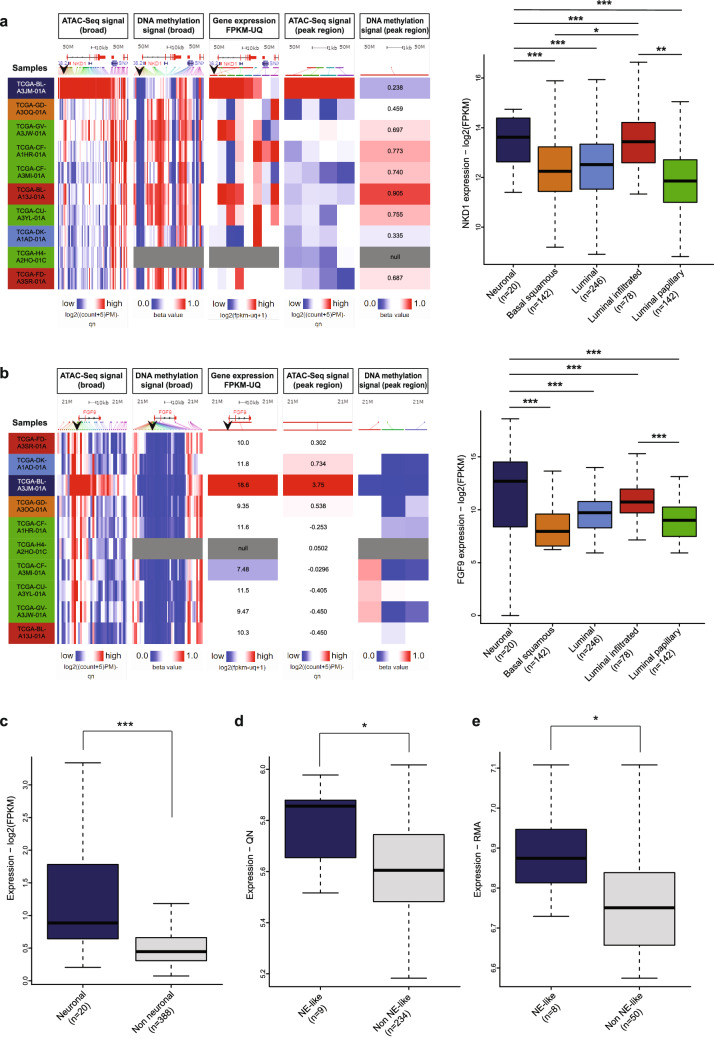
(Epi)genomic profiles of ß-catenin target genes regulated by neuronal regulatory regions. a-b Xena Browser snapshots show the ATAC-seq, DNA methylation and gene expression profiles for the bladder cancer patients (n = 10) at *NKD1* (**a**) (Anova *p* value = 2.33e-16) and *FGF9* loci (**b**) (Anova *p* value < 2e−16). PostHocTest significance codes:*p < 0.05; **p < 0.01; ***p < 0.001. Regions marked by blue arrows indicate the neuronal regulatory element regulating the respective genes. In the snapshots left, the last two panels show the ATAC-seq and DNA methylation signals directly at the regulatory elements marked with the blue arrows. Boxplots next to the snapshots show the expression levels of *NKD1* and *FGF9* across the bladder cancer subgroups. (**c**–**e**) Expression of all ß-catenin target genes regulated by neuronal regulatory elements between neuronal and non neuronal subgroups of bladder cancer belonging to TCGA cohort^7^(c) (Wilcoxon rank sum test *p* value = 9.807e−07) and Neuroendocrine like (NE-like) and non NE-like bladder cancers^18^ belonging to the cohorts Sjödahl^17^ (**d**) (Wilcoxon rank sum test *p* value = 0.01959) , Iyer^19^ (**e**) (Wilcoxon rank sum test *p* value = 0.0127) respectively. (*p < 0.05; **p < 0.01; ***p < 0.001).

As our transcription factor analysis associated ß-catenin-TCF/LEF factors with neuronal regulatory regions, we analyzed the (epi)genomic and transcriptomic profiles of ß-catenin targets in more detail. Inspecting the regulation of *NKD1* gene, which is a negative regulator of WNT signaling and has increased expression in several cancers with aberrant WNT signaling^35^, showed the hypomethylation at the regulatory element targeting this gene. Furthermore, analyzing the gene expression data available for 408 samples, we were able to show that *NKD1* was significantly upregulated in neuronal subtype (*p* value = 2.33e−16) (Fig. 4a). Similarly, checking the (epi)genomic profiles at *FGF9* locus, highly implicated in neuronal differentiation^36,37^ showed the hypomethylation directly at the regulatory element targeting this gene, with significantly increased patterns of expression in the neuronal subtype (Fig. 4b). Furthermore, we discovered that expression patterns of ß-catenin target genes were highly correlated in bladder cancer. Expression of *NKD1* and *NOTUM*, which is also a negative regulator of WNT signaling^38,39^, were significantly correlated (r = 0.53), where samples belonging to the neuronal subtype mark the corner with highest expression patterns (see Supplementary Fig. S4 online). Lastly, expression profiles of all genes which were targets of ß-catenin and regulated by neuronal regulatory elements revealed a significantly high expression of these genes in neuronal subtype compared to other subtypes (Fig. 4c)*.* To validate our findings with respect to activation of ß-catenin targets in neuronal subtype, we used gene expression data from other bladder cancer cohorts in addition to TCGA. Recently, the study by^18^ analyzed 1750 bladder cancer transcriptome and annotated molecular subtypes for these samples. The molecular subtypes defined in this study included a subgroup defined as ‘neuroendocrine-like’. We accessed the transcriptome data from the additional two cohorts included in^18^ and analyzed the expression profiles of β-catenin target genes regulated by neuronal active regulatory elements we identified in this study. Our analysis showed that the same set of genes were significantly upregulated in neuroendocrine-like classified bladder cancer in these two additional cohorts (Fig. 4d,e, see Supplementary Table S5 online), in line with our findings with the TCGA cohort. Together, our findings suggest the upregulation of the genes involved in WNT signaling via activation of ß-catenin in neuronal subtype bladder cancer.

### Expression of ß-catenin targets highly correlate with expression of N-cadherin

**Figure 5:**
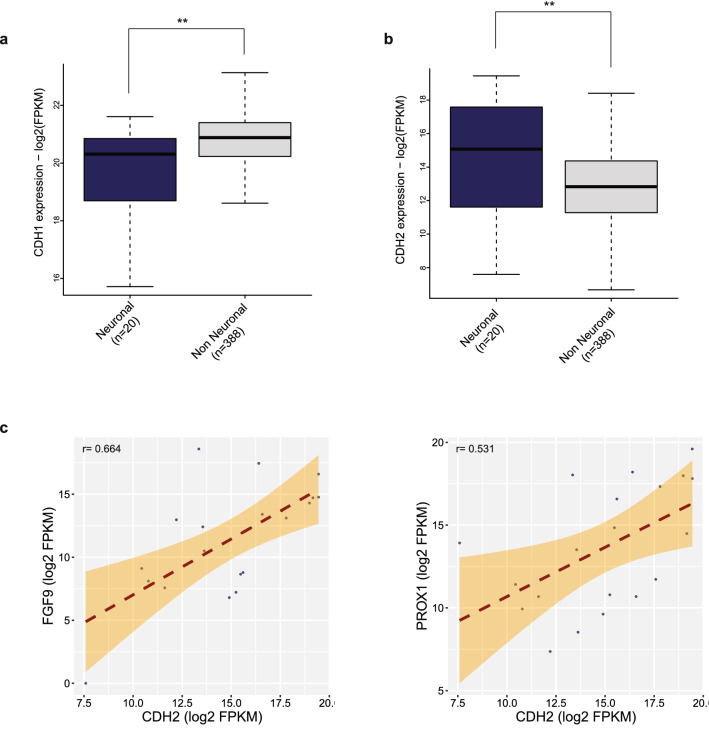
Expression of cadherins and their relation to ß-catenin target gene expression. a-b Boxplots represent the expression of N-cadherin (**a**) (Wilcoxon rank sum test *p* value = 0.0083) and E-cadherin (**b**) (Wilcoxon rank sum test *p* value = 0.010) in neuronal and non-neuronal bladder cancer patients. (**c**) Scatter plots compare the expression of N-cadherin with the ß-catenin targets regulated by neuronal regulatory elements, *FGF9* (Pearson correlation test *p* value = 0.001391) and *PROX1* (Pearson correlation test *p* value = 0.01583). (*p < 0.05; **p < 0.01; ***p < 0.001).

It is known that loss of E-cadherin expression and increase of N-cadherin expression, so called the ‘cadherin switching’ are the hallmarks of epithelial to mesenchymal transition in cancer^40^. In this context, N-cadherin expression was previously shown to be a prognostic marker in bladder cancer where its highest expression is detected in the stage T2 and T3 tumors^41^. Comparing the expression levels of N-cadherin and E-cadherin revealed significantly higher expression of N-cadherin and significantly lower expression of E-cadherin in neuronal bladder cancer compared to non-neuronal samples (Fig. 5a,b). It is also known that activation of WNT signaling drives the expression of N-cadherin while downregulating the expression of E-cadherin^42^. At this point, we wanted to see whether there is any association between N-cadherin expression and activation of ß-catenin/WNT signaling. Our analysis revealed significant correlations between N-cadherin expression and expression of ß-catenin target genes regulated by neuronal specific regulatory elements and involved in neurogenesis (Fig. 2a), such as *FGF9 and PROX1* (Fig. 5c) in the samples belonging to the neuronal subtype while doing a similar correlation analysis with E-cadherin expression, we detected almost no significant correlation (see Supplementary Fig. S5 online). To the contrary, performing the same analysis for non-neuronal bladder cancer patients, the correlations between N-cadherin and ß-catenin targets were almost close to zero (see Supplementary Fig. S5 online). Overall, these results suggest that high expression of N-cadherin is associated with the activation of ß-catenin/WNT signaling in neuronal bladder cancer, potentially influencing the invasive properties of this subtype.

### Neuronal bladder cancer patients are mutant for ß-catenin and ß-catenin destruction complex components

**Figure 6:**
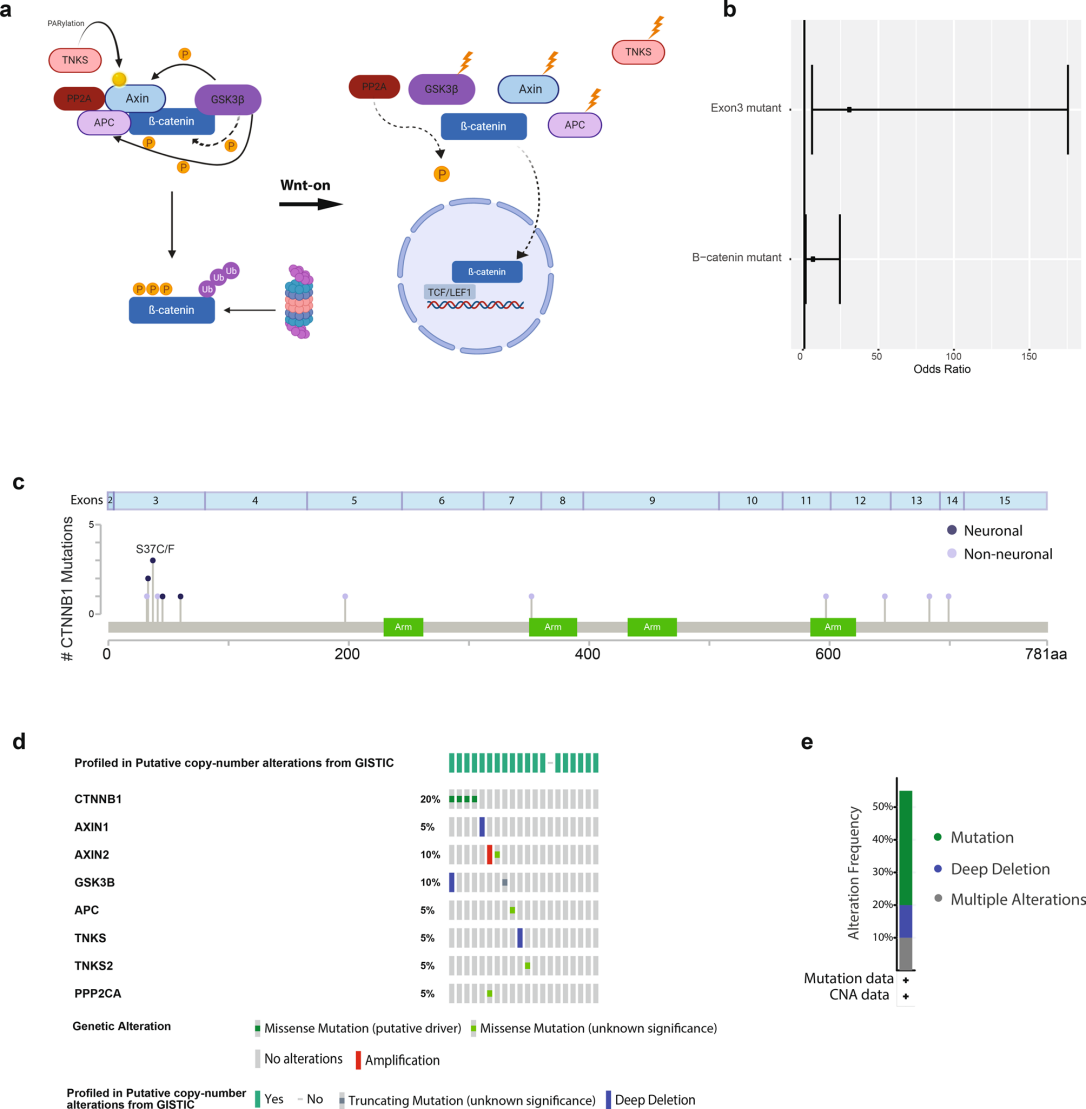
Mutation profiles of ß-catenin and ß-catenin destruction complex components in bladder cancer patients. (**a**) Cartoon illustrating ß-catenin and ß-catenin destruction complex. (Created with BioRender.com) (**b**) Plots show the odds ratio (with 95% confidence interval) demonstrating the enrichment of all ß-catenin and exon 3 ß-catenin mutations in neuronal bladder cancer. (**c**) Lollipop plot showing the mutations of ß-catenin in bladder cancer (neuronal:dark blue and non-neuronal:gray). (**d**) Oncoprint image displaying the mutation profile of ß-catenin and ß-catenin destruction complex components in samples belonging to the neuronal subtype. (**e**) Bar plot showing the cumulative mutation rate ß-catenin and ß-catenin destruction complex components.

ß-catenin, being as the core downstream component of the WNT signaling, its nuclear localization is subject to a tight control via the ß-catenin destruction complex. In the absence of WNT, ß-catenin is phosphorylated and degraded by the destruction complex and in the presence of WNT, ß-catenin translocates to the nucleus and activates ß-catenin-TCF/LEF target genes^43^ (Fig. 6a). As we identified neuronal regulatory regions to be associated with the ß-catenin-TCF/LEF complex, we wondered whether dysregulation of ß-catenin might have an effect on the aberrant activation of WNT signaling in neuronal subtype of bladder cancer. First, we checked the mutation status of ß-catenin in all bladder cancer patients. Our findings revealed a 20% mutation rate of ß-catenin in neuronal subtype compared to 4.34% mutation rate in non-neuronal samples. This result showed significant mutation rate of ß-catenin in neuronal bladder cancer (Fisher’s exact test *p* value = 0.0142) (Fig. 6b), which was a finding not reported before. In literature, it was previously shown that mutations in ß-catenin occurring on exon 3 are oncogenic and cause nuclear accumulation of ß-catenin^44^. At this point, upon classifying ß-catenin mutations in all bladder cancer patients, we discovered even a more significant association of exon 3 ß-catenin mutations with the neuronal subtype (odds ratio = 31.33, *p* value = 1.786e-05) (Fig. 6b,c, see Supplementary Fig. S6 online). Importantly, all the ß-catenin mutations identified for neuronal samples were damaging and present in COSMIC database^45^, and result in loss of phosphorylation sites, critical for the degradation of ß-catenin. Next, upon extending the mutation analysis for bladder cancer samples belonging to neuronal subtype for other proteins which are part of ß-catenin destruction complex (Fig. 6a), we identified that cumulatively 55% of all neuronal samples had mutations in ß-catenin itself or in the destruction complex components (Fig. 6d,e). The algorithms we used predicted the majority of these mutations occurring in the components of the ß-catenin destruction complex to have a damaging effect on the protein function (see Supplementary Table S6 online). Collectively, our findings strongly argue that mutations in ß-catenin itself or its destruction complex components result in aberrant accumulation of ß-catenin in nucleus and activation of WNT signaling as a result of upregulation of ß-catenin target genes.

## Discussion

In this study, starting from the re-analysis of ATAC-seq data generated for 10 MIBC samples, we were able to identify three regulatory clusters for MIBC. As there were only 10 MIBC samples with available ATAC-seq data, the regulatory clusters we defined in this study may not be final or definite but provided relevant regulatory characteristics of MIBC. Recently, the study by Kamoun et al. 2020 classified the MIBC into its molecular subgroups using transcriptome data over 1700 MIBC specimens, including also the same specimens from the TCGA cohort we analysed in this study^18^. Inspecting the classification from this new study showed that the TCGA subgroup annotations of the 10 MIBC were highly comparable with the subgroup annotations from^18^ (see Supplementary Table S7 online), suggesting for the applicability of the regulatory region classifications we determined.

With a specific focus on the neuronal cluster we identified, our analysis revealed important insights about the regulation of neuronal bladder cancer. Although there was only one sample belonging to neuronal molecular subtype^7^ with available ATAC-seq data, in depth integration of our findings based on the neuronal regulatory regions we defined with gene expression and DNA methylation data available over 400 MIBC from the same TCGA cohort proved the competence of the regulatory region definitions we constituted (Fig. 2). These results emphasize the potential of using regulatory genomics data in understanding of transcriptional networks characterizing tumor subtypes.

Linking the three regulatory clusters we defined with their target genes enabled us to decipher the pathways differentially regulated in bladder cancer. The genes which were targets of non-neuronal regulatory elements were mainly involved in epithelial differentiation and related pathways. This finding might suggest that neuronal bladder cancer diverges from the non-neuronal ones by the lack of epithelial cell features. Surprisingly, we identified a cluster of regulatory elements which showed activity only in one luminal papillary sample (luminal outlier cluster). Further following upon this finding, we identified this sample to receive Carboplatin and Gemcitabine (Gemzar) combined chemotherapy, which might be influential on the regulatory characteristics of this sample among the other luminal papillary samples.

Undoubtedly, among the three regulatory clusters we defined, neuronal cluster was the most interesting. We determined the target genes of this cluster to be associated primarily with the WNT signaling. Afterwards, further identification of the ß-catenin and TCF/LEF motifs enriched at the neuronal regulatory elements and enrichment of ß-catenin mutations completed the circle explaining the molecular regulation of neuronal bladder cancer by WNT signaling. Within the context of the ß-catenin mutations we identified in bladder cancer, an exceptional enrichment for exon 3 ß-catenin mutations was remarkable. These mutations were, previously referred as highly ‘oncogenic’^44,46^, and identified to disrupt of β-catenin degradation and cause its nuclear accumulation in the presence of phosphorylation site mutations S33, S37 and S45^47–49^, which are the same mutations we identified in neuronal bladder cancer. Therefore, these prior knowledge and our findings highly argue for the role of ß-catenin/WNT signaling in neuronal subtype.

Having identified WNT signaling and ß-catenin mutations to be associated with neuronal bladder cancer, we wondered about the role of WNT signaling in ‘neuronal’ character of this subtype. From literature, it is known that WNT signaling regulates self-renewal and differentiation of neuronal precursor cells^50,51^. Recent studies also showed that small molecules inducing WNT signaling enhance the neuronal differentiation of embryonic stem cells^52^ and human urine cells can be converted to neurons using a combination of small molecules, including WNT activator^53^. In our study, it appears that among the target genes of neuronal regulatory elements which are also ß-catenin targets (results of EnrichR), there are several genes involved in neurogenesis, including *FGF9* and *PROX1*. These results suggest aberrant activation of ß-catenin in neuronal bladder cancer as a result of the mutations in ß-catenin/ß-catenin destruction complex components ultimately activating WNT signaling and enhancing neuronal differentiation.

Previously, it was also shown that expression of many WNT antagonists were upregulated in hepatoblastomas^54^ and murine lenses^55^ which were mutant for ß-catenin and this pattern is regarded as an indication of activation of the WNT signaling. In our analysis, identification of active neuronal regulatory elements targeting negative regulators of WNT signaling such as *NKD1* and *NOTUM* and significantly higher expression of these genes (Fig. 4a, see Supplementary Fig. S4 online) argues for a similar phenomenon occurring in neuronal subtype of bladder cancer as a result of mutations in ß-catenin.

According to the molecular subtypes defined in the latest TCGA study^7^, neuronal subtype is the most aggressive one and it has the worst survival rate among the other subtypes. Importantly, 17 out of 20 samples belonging to neuronal subtype in the same TCGA study did not show any neuroendocrine origin, suggesting for the convergence of molecular mechanisms in appearance of regulatory signatures characteristic of this subtype. Despite the lowest survival rates, there are not yet available specific treatment options for neuronal bladder cancer. Current treatment regimens mostly consist of neoadjuvant chemotherapy combined with radical cystectomy or radiotherapy^56^. Thus, development of specific treatment strategies for neuronal type bladder cancer is essential. Our results highly argue for the activation of WNT signaling in neuronal subtype via ß-catenin mutations. Furthermore, validation of our findings via the gene expression data of two additional cohorts^17,19^ in addition to the TCGA cohort strengthens our findings with regard to activation of ß-catenin target genes in neuronal subtype bladder cancer. This hypothesis can be tested using experimental approaches, for instance via direct testing of the ß-catenin activation on solid tumor specimens. Therefore, we suggest that development of therapies which target ß-catenin might be highly beneficial for the patients belonging to neuronal subtype bladder cancer. In fact, there are several therapeutics developed and which are in different phases of clinical trials against ß-catenin itself or the components of the ß-catenin destruction complex for the treatment of colorectal cancer^57^, pancreas cancer, and several other solid tumors^58^.

## Conclusions

Our thorough analysis of active chromatin regions defined for bladder cancer and its integration with DNA methylation and gene expression data available for bladder cancer highlights the damaging mutations of ß-catenin in neuronal subtype of bladder cancer, and associates these mutations with activation of WNT signaling in the neuronal subtype.

## Supplementary information

Supplementary Information 1.

Supplementary Table S1.

Supplementary Table S2.

Supplementary Table S3.

Supplementary Table S4.

Supplementary Table S5.

Supplementary Table S6.

Supplementary Table S7.

## Data Availability

TCGA ATAC-seq, DNA methylation, gene expression and mutation data for the bladder cancer used in this study is freely available at https://portal.gdc.cancer.gov/ and does not require any specific permissions.
