# Supplementary material for: Analysis of open chromatin regions in bladder cancer links β-catenin mutations and Wnt signaling with neuronal subtype of bladder cancer
Source: Sci Rep. 2020 Oct 29;10:18667. doi: 10.1038/s41598-020-75688-0 (PMC7596510; doi:10.1038/s41598-020-75688-0)
Supplement: Supplementary file 1 — Supplementary Information 1. [file 41598_2020_75688_MOESM1_ESM.pdf]

Analysis of open chromatin regions in bladder cancer links β-catenin mutations and Wnt signaling with neuronal subtype of bladder cancer

Supplementary Information

Aleyna Eray[1,2], Perihan Yağmur Güneri[1,2], Gülden Özden Yılmaz[1], Gökhan Karakülah[1,2], Serap Erkek[1,]

[1] Izmir Biomedicine and Genome Center, Inciralti 35340, Izmir, Turkey

[2] Dokuz Eylül University Izmir International Biomedicine and Genome Institute, Inciralti 35340, Izmir, Turkey

Manuscript correspondence:

Serap Erkek

Research Group Leader

(Epi)genomics of Cancer Group

Izmir Biomedicine and Genome Center

Dokuz Eylül University Health Campus

Mithatpaşa Caddesi No:58/5

35330 Balçova, İzmir, Turkey

Phone: +90(232) 299 41 67

Supplementary Fig. S1. Analysis of the DNA methylation levels in luminal outlier regulatory elements.

Supplementary Fig. S2. Kegg pathway analysis for the target genes of the luminal outlier regulatory elements.

Supplementary Fig. S3. HOMER de novo transcription factor motif analysis at the neuronal regulatory regions.

Supplementary Fig. S4. Correlation between the expression of ß-catenin target genes NKD1 and NOTUM in neuronal and non-neuronal bladder cancer.

Supplementary Fig. S5. Expression of E-cadherin and N-cadherin and their relation to the expression of ß-catenin targets in neuronal and non-neuronal bladder cancer.

Supplementary Fig. S6. Contingency tables of ß-catenin mutation status of neuronal and non-neuronal bladder cancer samples.

Supplementary Table S1. The genes linked to the regulatory elements of the three clusters, corresponding peak names and their coordinates.

Supplementary Table S2. Chemotherapy drugs used for the treatment of samples and their TCGA subgroups.

Supplementary Table S3. Complete ChEA results reported by EnrichR program obtained for the target genes of neuronal regulatory elements.

Supplementary Table S4. Top 10 transcription factor motifs and their enrichments at neuronal regulatory elements as reported by HOMER.

Supplementary Table S5. Expression values of the ß-catenin target genes in three different cohorts (TCGA, Iyer, Sjödahl) and their corresponding molecular subgroups.

Supplementary Table S6. Functional annotation of the mutations identified for ß-catenin destruction complex components in neuronal bladder cancer.

Supplementary Table S7. TCGA and Kamoun subgroup annotations for the 10 MIBC samples.

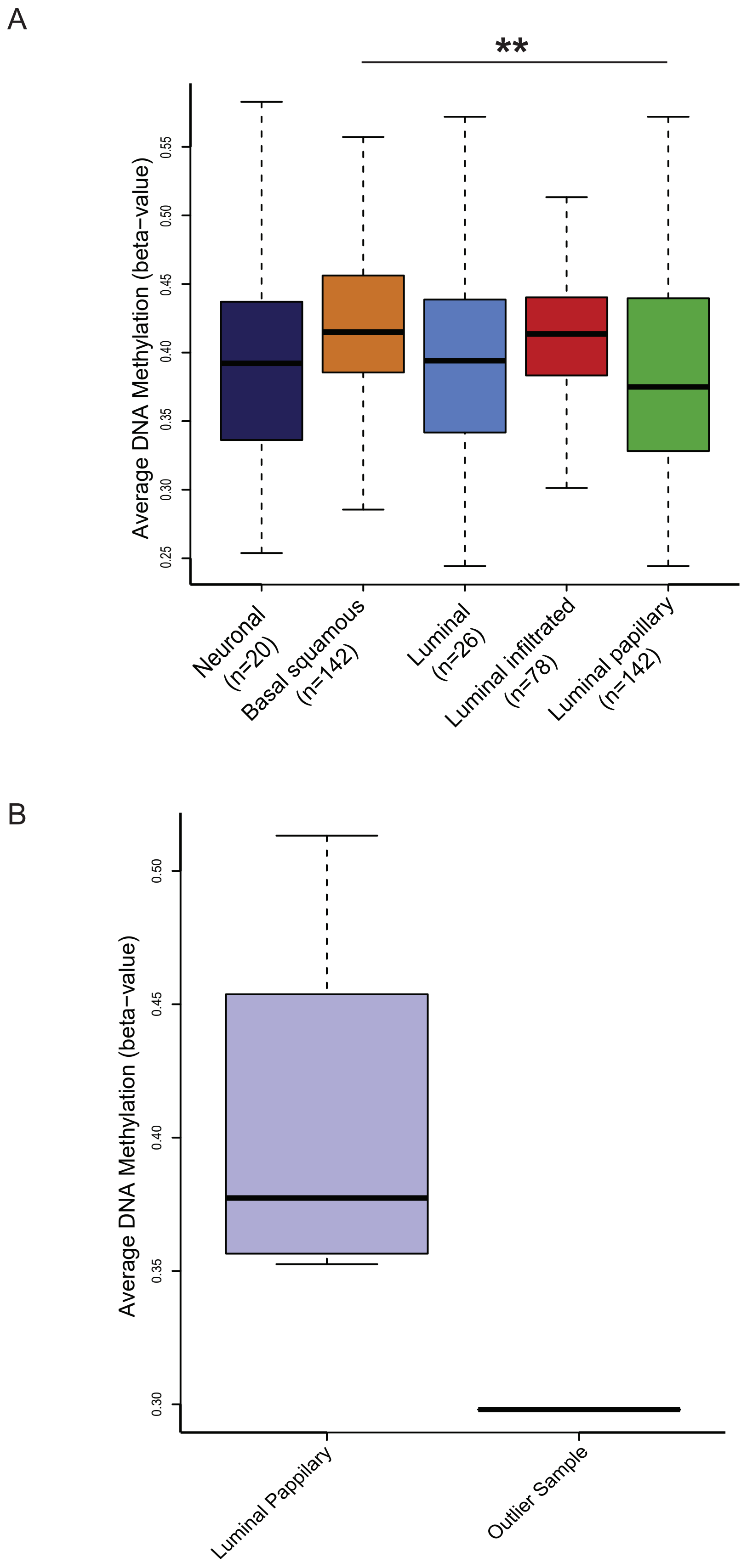

Supplementary Fig. S1. Analysis of the DNA methylation levels in luminal outlier regulatory elements. a Boxplot shows the DNA methylation levels (Beta values) of mRNA subgroups at the luminal outlier regulatory regions (cluster 3) (*p<0.05; **p<0.01; ***p<0.001).

b Boxplot displays the DNA methylation levels of luminal outlier sample (n=1) and other luminal papillary samples (n=4) at the cluster 3 regulatory regions.

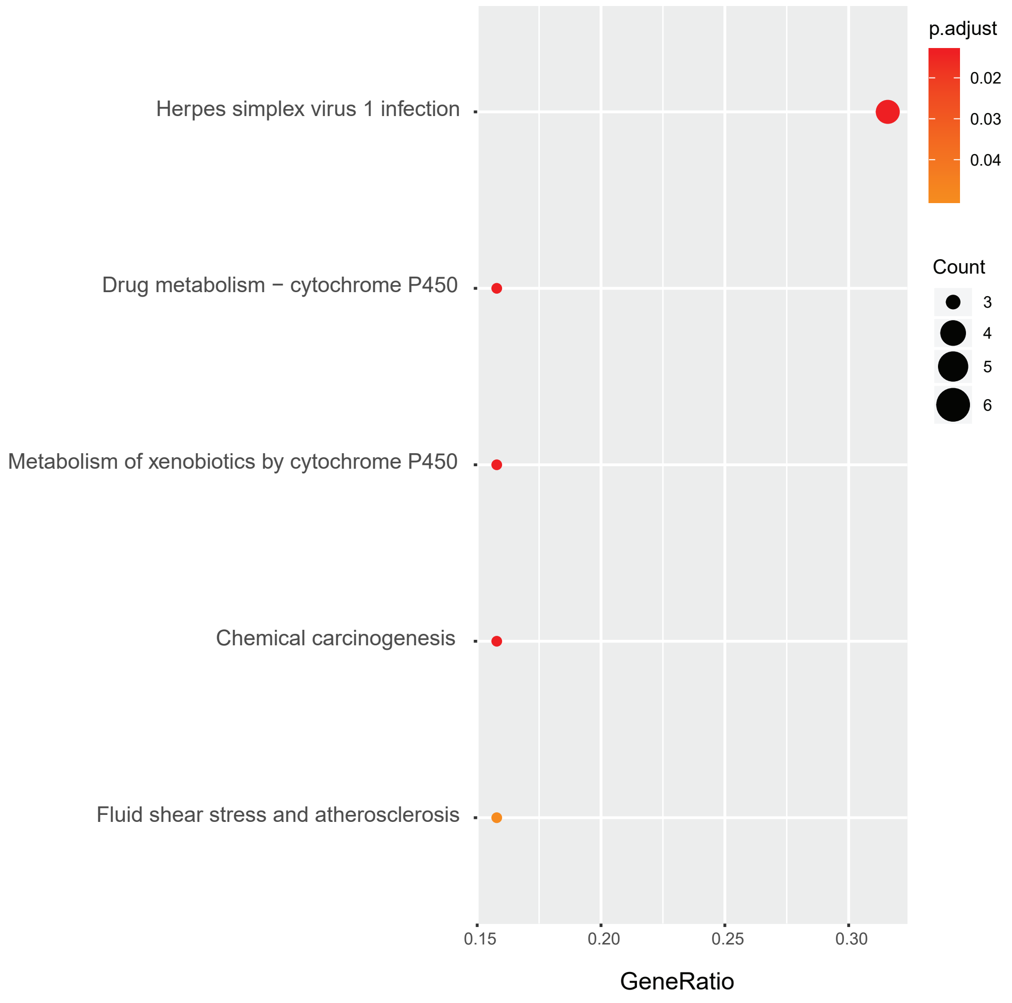

Supplementary Fig. S2. Kegg pathway analysis for the target genes of the luminal outlier regulatory elements. Dot sizes indicating gene counts of specific terms.

Motif	Transcription Factor	p-value
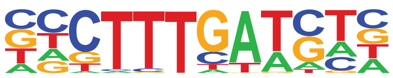	LEF1	1e-690
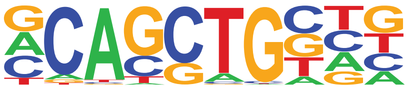	E2A	1e-140
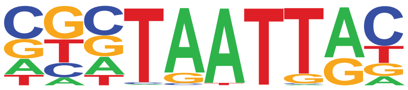	Dlx2	1e-137

Supplementary Fig. S3. HOMER de novo transcription factor motif analysis at the neuronal regulatory regions. Top three enriched motifs and best matched transcription factors are displayed.

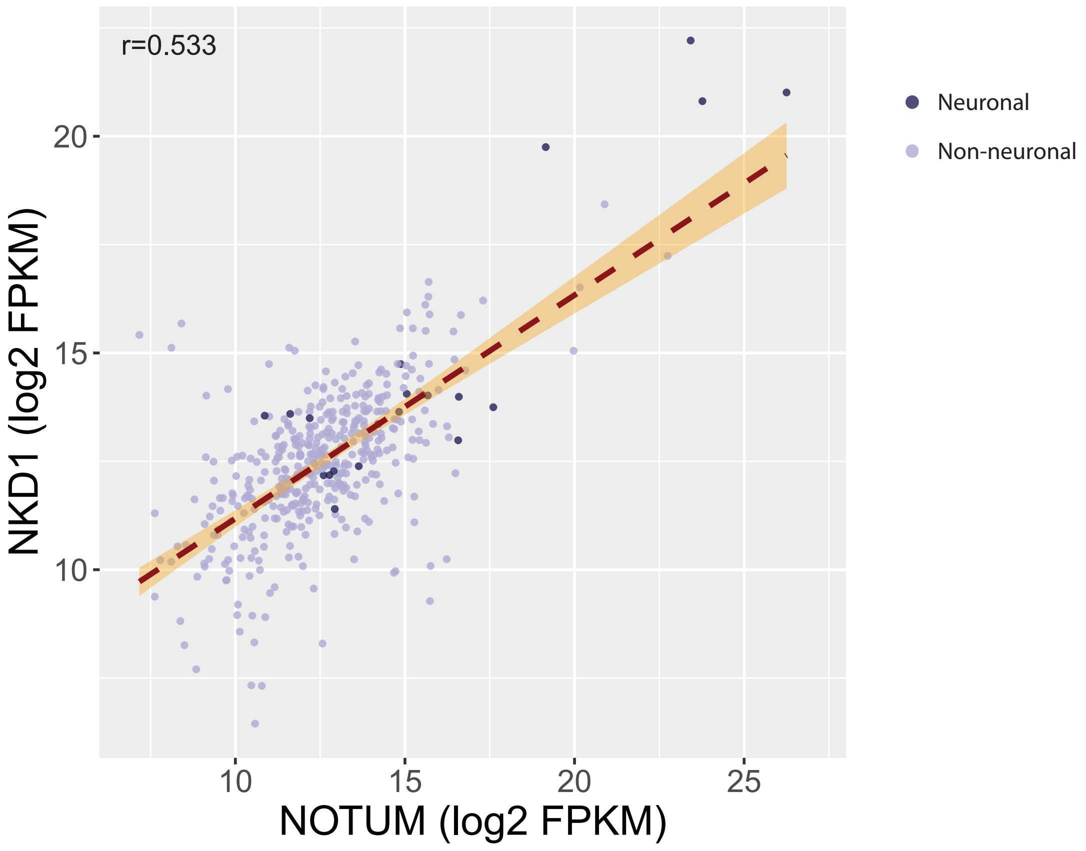

Supplementary Fig. S4. Scatter plot showing the correlation between the expression of ß-catenin target genes NKD1 and NOTUM. (r=0.53)

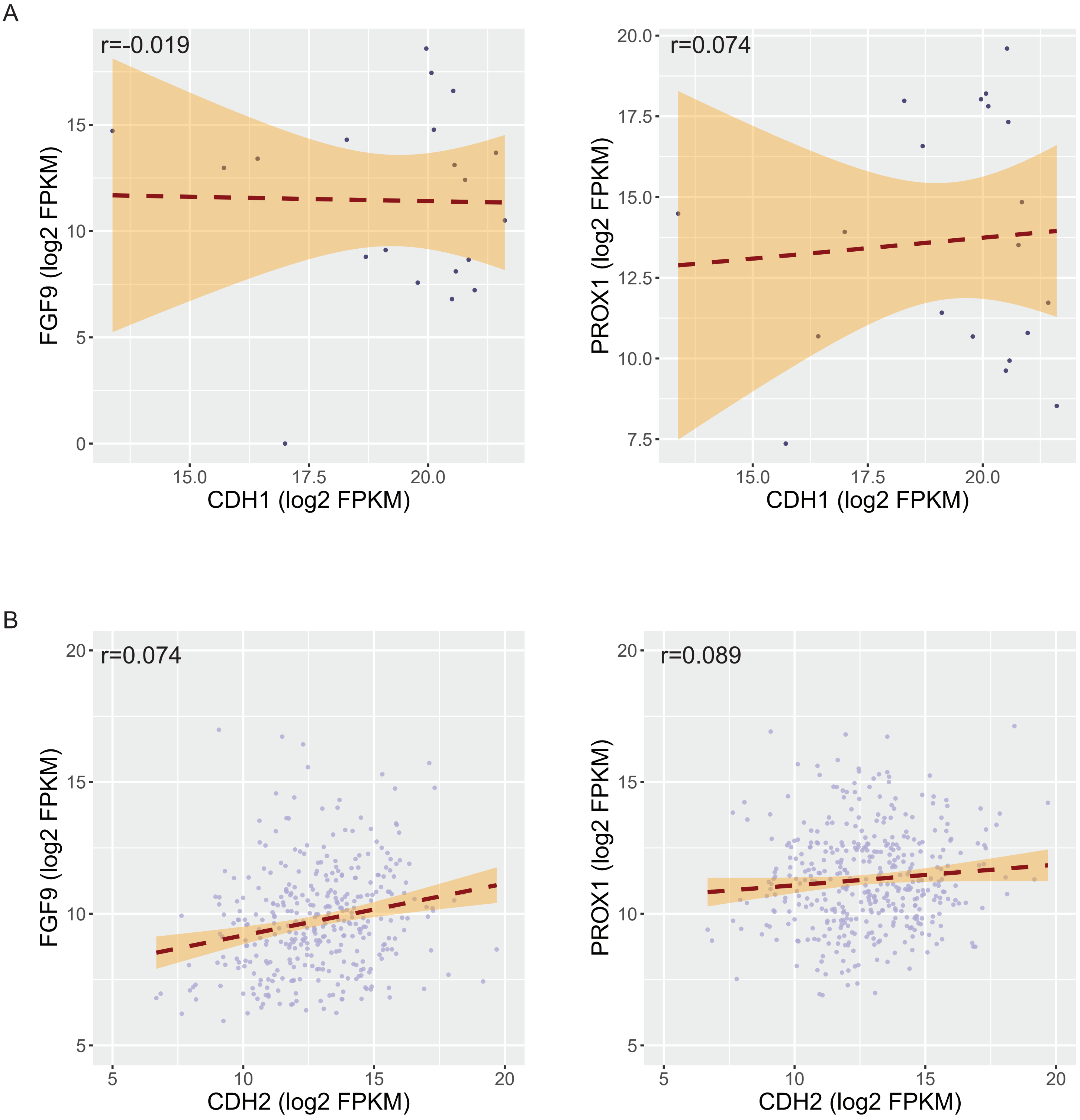

Supplementary Fig. S5. Expression of E-cadherin and N-cadherin and their relation to the expression of ß-catenin targets in neuronal and non-neuronal bladder cancer. a Scatter plot compares the expression of E-cadherin with the expression FGF9 and PROX1 in neuronal bladder cancer. **b** Scatter plot compares the expression of N-cadherin with the expression FGF9 and PROX1 in non-neuronal bladder cancer.

A

Group	ß-catenin mutant	ß-catenin non-mutant
Neuronal	5	15
Non-neuronal	17	375

B

Group	ß-catenin exon3 mutant	ß-catenin exon3 nonmutant
Neuronal	5	15
Non-neuronal	4	388

Supplementary Fig. S6. Contingency tables of beta-catenin mutation and beta-catenin exon 3 mutation only. The mutation status of the TCGA bladder cancer samples was determined through the cBioPortal database. **a** Table shows beta-catenin mutation status of TCGA bladder cancer samples. **b** Table shows beta-catenin exon 3 mutation status of the samples.
